# Longitudinal investigation of the factor structure of the Parkinson's disease activities of daily living, interference and dependence instrument

**DOI:** 10.3389/fneur.2022.941788

**Published:** 2022-09-23

**Authors:** Cristian Sirbu, Brian K. Saxby, Cynthia W. McNamara, Linda S. Deal

**Affiliations:** ^1^Cronos Clinical Consulting Services, Inc., Lambertville, NJ, United States; ^2^Charleston Area Medical Center Institute for Academic Medicine, Charleston, WV, United States; ^3^West Virginia University School of Medicine Charleston Division, Charleston, WV, United States; ^4^Patient-Center Outcomes Assessment, Pfizer, Inc., New York, NY, United States

**Keywords:** Parkinson's disease, activities of daily living, PD-AID, patient reported outcomes (PRO), longitudinal validation, Multilevel Factor Analysis

## Abstract

The Parkinson's Disease Activities of Daily Living, Interference, and Dependence Instrument© (PD-AID) is a patient-reported outcome (PRO) instrument, recently developed to assess the clinical benefit of Parkinson's Disease (PD) treatment. The PD-AID consists of morning and evening assessments, administered daily. To benefit from the full set of the repeated observations over time, analytic approaches that account for both within- and between-individual variability are required. The current study aimed to employ the advantages of exploratory Multilevel Factor Analysis (MFA) on data collected from 93 participants with moderate to advanced PD, currently using and responding to Levodopa (L-Dopa), who completed the PD-AID twice daily as part of a prospective, non-intervention, observational study for ~28 days. Average daily completion rates were comparable for the Morning and the Evening PD-AID (78% and 74%, respectively). The intraclass correlation coefficients for the Morning and Evening PD-AID items were in the range of 0.70–0.90, with an average of 0.81 for the Morning PD-AID items and 0.83 for the Evening PD-AID items, suggesting that most variability (81%–83%) in responses was due to between-individual variability. For the Morning PD-AID, one factor (including nine out of 10 Morning PD-AID items) emerged at the between-individual level and four factors (core physical actions, basic self-care activities, feeding, and interference & dependence) at the within-individual level. For the Evening PD-AID, there were four between-individual factors (basic activities of daily living ADLs, life interference, impact on planning, and emotional consequences) and five within-individual factors (basic ADLs, toileting, life interference, medication planning, and emotional impact). The factors had high reliability.

## Introduction

Parkinson's disease (PD) is a chronic and progressive neurological disorder defined by symptoms of bradykinesia, rigidity, tremor, and postural instability (1). The disease affects about 1.5 million people in the United States (US) with the prevalence of PD expected to double by 2030 (2). Patients with PD experience significant impairment to their activities of daily living (ADLs) and increased reliance on caregivers during advanced stages. PD is frequently associated with motor symptoms caused by the loss of dopamine neurons in the basal ganglia and the substantia nigra. Levodopa (L-Dopa), an exogenous source of a dopamine precursor, remains the most effective and widely used pharmacotherapy for PD; however, its prolonged use is characterized by fluctuations in efficacy and disabling dyskinesias that negate its beneficial effects and are difficult to treat (3). Fluctuations in efficacy of L-Dopa are characterized by periods of ON response (when motor fluctuations are controlled) and OFF response (when motor fluctuations are not controlled). Because of the duration of dose effect with L-Dopa, the OFF response is more prevalent upon waking due to treatment response wearing off during the night. This leads to an impact on ADLs immediately upon waking which is reported to be different from the impact on ADLs during the rest of the day (4).

The Parkinson's Disease Activities of Daily Living, Interference and Dependence Instrument© (PD-AID) is a patient-reported outcome (PRO) instrument developed to assess the clinical benefit of PD treatment from the patient perspective. Specifically, it targets concepts that give meaning to PD motor symptoms in terms of their impact on everyday life. Details of its development and content validation have been published previously (5). The PD-AID was developed in accordance with the US Food and Drug Administration's PRO guidance to overcome the limitations of existing instruments. In particular, the PD-AID content was selected based upon direct input from individuals living with PD (6). The PD-AID focuses on direct and proximal consequences to day-to-day functioning resulting from PD motor fluctuations as well as areas of unmet priority related to treatment. Specifically, it assesses relevant ADLs, dependence on others to perform ADLs, and life interference due to accommodating PD symptoms and treatment. The intended context of use for the PD-AID is as a clinical trial efficacy outcome evaluating clinical changes from the perspective of individuals living with moderate-to-advanced PD who are experiencing motor fluctuations.

The PD-AID addresses important considerations in the process of patient-centered scale development identified by the Movement Disorder Society (MDS) Technology Task Force and MDS Rating Scales Program Electronic Development Ad-Hoc Committee (7). An important concern with daily diaries is the failure to use data fully in their psychometric evaluation. The use of average scores over multiple days for factor analysis, neglects the daily variability in patients' symptoms (8, 9).

Multilevel Factor Analysis (MFA) is an analytic approach that allows the structural analysis of longitudinal data taking into account the within- and between-individual variability (10, 11). Because daily diaries include repeated observations for each individual participant, the score variance derived from diary data includes two components [within (intra-) and between (inter-) individual variability]. MFA allows separation of these two sources and the fitting of latent variables (factors) for both the within- and between-individual variability. Factor analyses of longitudinal diaries (i.e., daily entries) averaged across multiple days are based on the assumption that the within- and between-individual factor structures are identical. However, as indicated by Molenaar, the structures of within- and between- individual covariations are different in a majority of cases (12). Further, in rare situations when those structures are the same, the item contributions (loadings) to within- and between-individual factors are different.

The current study had two aims: (1) to identify the factorial structure of PD-AID based on exploratory MFA using data collected in a non-interventional study of PD patients who were instructed to complete the measure twice daily for a minimum of 28 days; and (2) to evaluate the compliance with completion of the PD-AID. Once established with consideration for both the cross-sectional and longitudinal dimensions of the data, the factor structure will provide the basis for subsequent efforts to develop evidence-based scoring algorithms for the PD-AID using Item Response Theory.

## Methods

Analyses were conducted on data collected from electronic administration of the PD-AID as part of a prospective, non-interventional observational study of 93 patients with moderate-to-advanced PD currently using and responding to L-Dopa who experienced motor fluctuations. Details on the study procedures can be found in Deal et al. (13). The primary objective was to gather fit-for-purpose evidence for the use of the PD-AID in defining endpoints to support labeling claims. Data collection and study procedures were conducted in accordance with the Declaration of Helsinki ethical principles, the principles of Good Clinical Practice, and regulatory requirements as applicable. De-identified data were used in the analyses.

### Participants

Participants were males and females between 45 and 85 years of age (inclusive), with a physician-confirmed diagnosis of PD, Hoehn & Yahr (H&Y) stage ≤ 3 as documented in medical records within the past year, and currently using and responding to a stable dose of L-Dopa ≥400 mg daily (14). This last requirement was subsequently revised to ≥300 mg daily when it became evident that recruitment was a challenge. Participation also required the ability to recognize L-Dopa “wearing off,” English fluency, and a willingness and ability to comply with all study instructions and scheduled visits. A history of surgical intervention for PD (e.g., deep brain stimulation), the presence of cognitive impairment or a psychiatric condition judged by the recruiting physician as interfering with the ability to complete questionnaires for 1 month, and current or planned (within the next two months) participation in an interventional PD clinical trial were causes for exclusion. Efforts were made to recruit participants from six US sites (California, Colorado, Florida, Missouri, New York and Washington) who were representative of the target clinical trial PD population for which the PD-AID was developed. This was accomplished by focusing recruitment efforts on obtaining an equal proportion of participants at H&Y stages 1, 2, and 3, as well as at least 20% experiencing dyskinesia.

### Parkinson's disease activities of daily living, interference and dependence instrument (PD-AID)©

The PD-AID consists of a set of items administered in the morning and a set of items administered in the evening. The Morning PD-AID comprises eleven items addressing whether the respondent required help performing core ADLs, including getting out of bed, walking inside the home, getting on or off the toilet, showering/bathing, grooming, dressing, preparing something to eat or drink, and feeding one's self. Each of these questions is gated to determine whether the individual was able to perform the activity on their own. If no help was needed, they are instructed to indicate the level of difficulty in performing the ADL on their own using a 5-point categorical response scale (CRS) ranging from “not at all difficult” to “extremely difficult.” If the activity was not performed that morning, the respondent is directed to indicate if the reason was “due to Parkinson's disease” or “other reason.” Additional items focus on whether their PD caused a delay in morning activities (Yes/No) and the degree to which PD influenced the respondents' level of dependence on others or interfered with getting ready for the day (using a 7-point CRS ranging from “not at all” to “completely interfered/dependent”). The Morning PD-AID takes ~3 min to complete. Respondents are asked to complete the Morning PD-AID after they have finished their morning routine to get ready for the day, but before lunchtime.

The Evening PD-AID consists of eighteen items addressing several core ADLs also included in the Morning PD-AID (walking around the house, using the toilet, preparing food, feeding oneself), ADLs not previously assessed (getting in or out of a vehicle, using an electronic touchscreen), and exploratory items. It takes about 5 min to complete and uses a recall of either “since completing your morning diary” (for the items assessed in the morning) or “in the past 24 hours” (for the remaining items). As in the Morning PD-AID, the items assessing ADLs are gated and use the same 5-point CRS. If the activity was not performed since completing the Morning PD-AID or in the past 24 hours, the respondent is directed to indicate whether the reason is “due to Parkinson's disease” or “other reason.” The exploratory items address PD interference with work (if employed) and leisure activities, as well as the need to plan daily activities around expectations related to PD treatment wearing off (e.g., prevent, delay, or cease activities). Respondents are instructed to complete the Evening PD-AID at the end of their day, ideally at the same time, before going to bed. For both the Morning and Evening PD-AID assessments, if a particular activity is done more than once during the given recall period, respondents are instructed to answer based on when it was at its worst or based on the instance when it was most difficult for them. Using branching, item responses are mapped to a transformed scale with 7 categories, where higher values indicate greater difficulty on ADL items, greater interference due to PD, or greater dependence on others (Supplementary Figure 1). The Morning and Evening PD-AID were completed daily for the duration of the study.

### Data analysis

Descriptive statistics were conducted using SAS version 9.4 (15) and the MFAs were conducted in MPlus version 8.5 (16). Descriptive statistics and floor/ceiling effects, defined as frequencies > 15% at the extremes of the response categories (17), were examined for each item and the proportion of missing data throughout the study was determined.

To determine the effect of missingness, data were analyzed in three steps (18). In the first step the dataset with missing data was analyzed using all the available data, based on the “pairwise present” Mplus default method. Next, five complete datasets were created in Mplus using multiple imputation based on the Markov chain Monte Carlo simulation utilizing the Gibbs sampling algorithm (the Mplus code for the development of multiple imputation datasets is provided in Supplementary material). Lastly, the analyses were replicated on each of the five complete datasets.

Intraclass correlation coefficients (ICC) were calculated for the Morning and Evening PD-AID to determine the measurement reliability. Reliability was assessed for a time window of four weeks. A similar analysis, averaging across seven days, was conducted using the first study week. ICC is defined as the amount of between-individual variability relative to total variability with larger ICC values reflective of greater between-individual differences.

Additionally, the ICCs informed the feasibility of conducting MFA. An ICC of zero indicates that all variation is due to within-individual differences in which case, MFA is not applicable. Once a non-zero ICC was established, the MFA was performed. Following the Muthen (19), and Grilli and Rampichini (20) approaches to MFA, the within-level covariation matrix was factor analyzed first using an unrestricted structure (saturated model with zero degrees of freedom) at the between-level. This was followed by a factor analysis of the between-level covariation matrix after restricting the number of within-level factors to the number determined in the previous step. The MPlus code used for the MFA analysis is provided in Supplementary material.

Exploratory MFA employed a weighted least-squares mean-adjusted estimator (WLSM) using a polychoric correlation matrix. Nested models were compared using the Satorra-Bentler scaled χ^2^ (Chi Square) difference test (21). The factors were allowed to correlate using the Geomin rotation. The following fit-indices were also calculated: root mean square error of approximation (RMSEA) with a value <0.05, standardized root mean-squared residual (SRMR) (within and between) with values < 0.08 and the comparative fit index (CFI) and Tucker-Lewis index (TLI) with values >0.95 for good fit (22). Factor reliability was evaluated using the McDonald's omega coefficient (23). Values higher than 0.70 indicate good factor reliability (24).

## Results

### Demographic and health information

Ninety-four participants enrolled in the study, although one participant did not provide any PD-AID data and was therefore excluded from the analyses. Demographics for the remaining 93 participants are shown in Table 1 (mean age 69 years; 68% male; 91% White). Using PD-specific data from the participants' medical records, clinicians reported 34 participants (37.6%) with dyskinesia associated with L-Dopa use. The mean time since PD diagnosis was 6.9 years (SD = 4.2; range 0.3–20 years).

**Table 1:** Participant-reported demographic and health information.

**Characteristic**	**Total sample** ***N* = 93**
Age, (years) Average (SD)	68.8 (8.6)
Gender, *n* (%)	
Male	63 (67.7%)
Female	30 (32.3%)
Race (all that apply selected), *n* (%)	
White	85 (91.4%)
Asian	4 (4.3%)
Black or African American	2 (2.2%)
American Indian or Alaska Native	1 (1.1%)
Not reported	1 (1.1%)
Ethnicity, *n* (%)	
Not Hispanic/Latino(a)	90 (96.8%)
Hispanic/Latino (a)	2 (2.2%)
Not reported	1 (1.1%)
Highest level of education, *n* (%)	
High school (no degree) or less	1 (1.1%)
High school graduate (or equivalent)	11 (11.8%)
Some college (no degree)	27 (29.0%)
Associate degree	7 (7.5%)
Bachelor's degree	28 (30.1%)
Master's degree	15 (16.1%)
Doctoral degree	4 (4.3%)
Work status (all that apply selected), *n* (%)	
Retired	60 (64.5%)
Working full-time	12 (12.9%)
On disability	9 (9.7%)
Working part-time	6 (6.5%)
Part-Time and Retired	2 (2.2%)
Homemaker	2 (2.2%)
Unemployed	1 (1.1%)
Other (reported as self-employed)	1 (1.1%)
General health status, *n* (%)	
Excellent	11 (11.8%)
Very good	25 (26.9%)
Good	47 (50.5%)
Fair	8 (8.6%)
Poor	2 (2.2%)
Means of assistance (if any), *n* (%)	
Spouse/partner	45 (48.4%)
Other family members	5 (5.4%)
Paid help	4 (4.3%)
Friends	2 (2.2%)
Spouse/Family/Friend/Paid	1 (1.1%)
Volunteer help	1 (1.1%)
Other (not specified)	34 (36.6%)
Not reported	1 (1.1%)
Hoehn and Yahr stage, *n* (%)	
Stage 1 (Unilateral symptoms only)	31 (33.3%)
Stage 2 (Bilateral symptoms; no balance or walking problems)	20 (21.5%)
Stage 3 (Problems with balance and walking)	42 (45.2%)

Six participants who had data for at least one administration of the PD-AID dropped out of the study for the following reasons: burdensome administration schedule (*n* = 4); difficulty entering answers into electronic device due to PD symptoms (*n* = 1) and difficulty understanding how to use the electronic device (*n* = 1).

### Summary analyses

Overall, during the 28 study days, the percentage of completion was 78% for the Morning PD-AID (mean missing = 22.3%, S.D. = 5.8%) and 74% for the Evening PD-AID (mean missing = 26%, S.D. = 3.7%) (Supplementary Figure 2). For item 7 (Working) of the Evening PD-AID, 64.8% of participants mentioned that they were not working due to reasons other than PD. The items distributions suggest a floor effect for all the Morning PD-AID items and most Evening PD-AID items (Supplementary Tables 1, 2).

### Assessment window for reliable measurement

All ICC coefficients for the entire study duration were higher than 0.70, indicating that measurement using the Morning and Evening PD-AID for a time window of at least four weeks is reliable. The ICC coefficients for the one-week assessment window were higher than 0.70 for every item except Evening PD-AID item 12 “Adjust medication schedule” (ICC = 0.63). The results suggest that the use of the Morning and Evening PD-AID is reliable when the items are averaged across 1 week (Supplementary Table 3).

### Multi-level factor analysis

**Figure 1:**
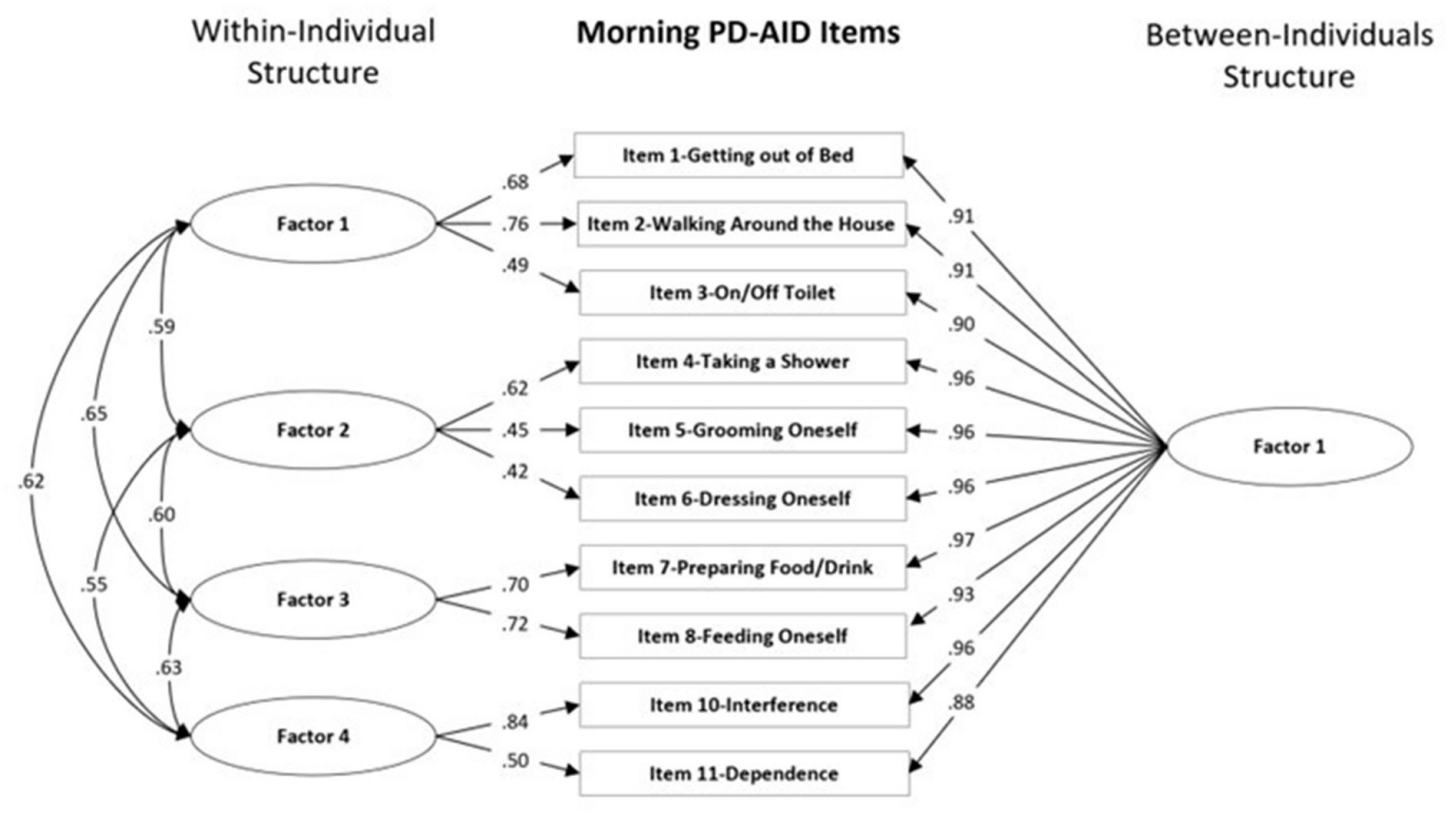
Within-individual and between-individuals factors standardized loadings and correlations for the Morning PD-AID.

**Figure 2:**
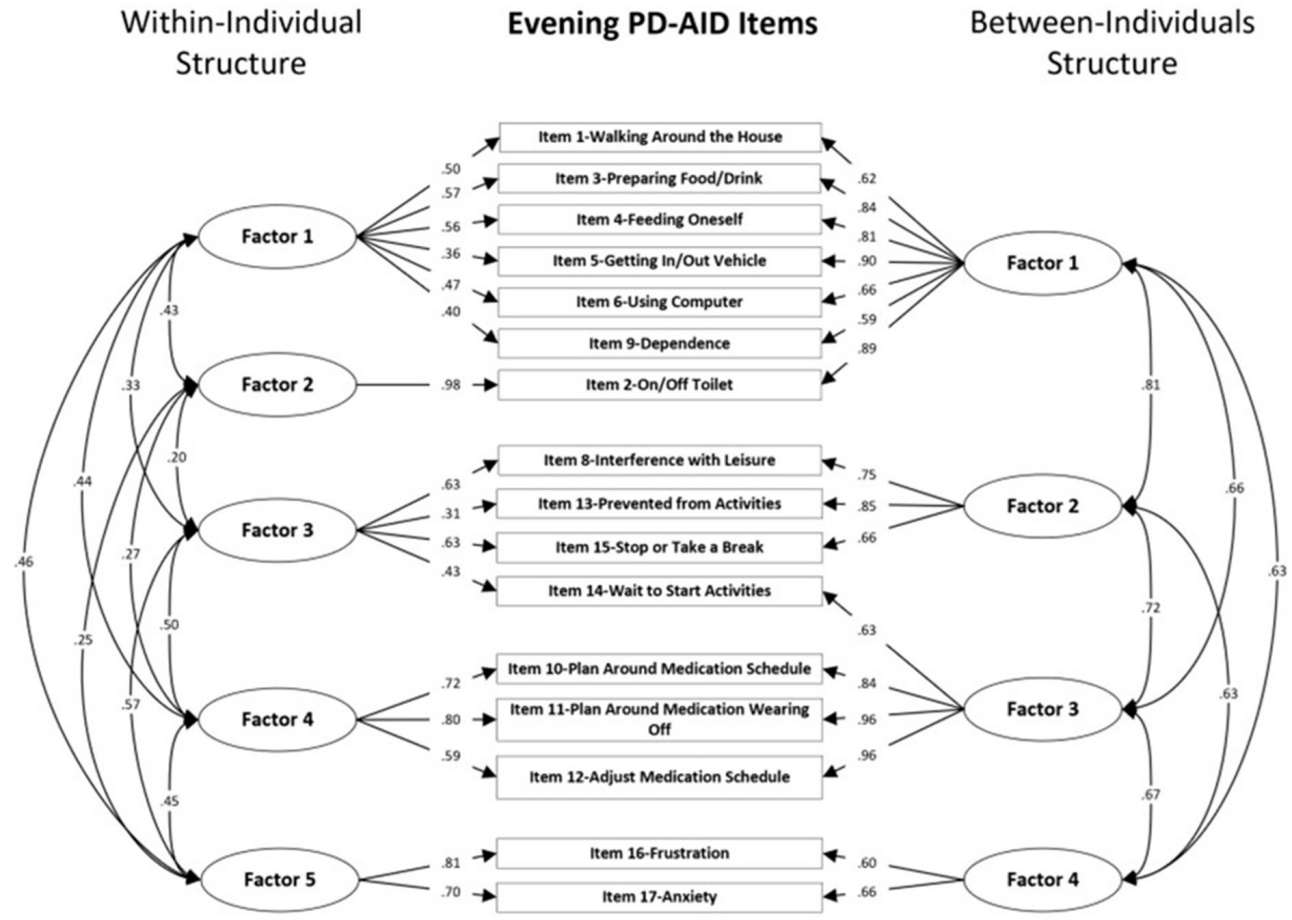
Within-individual and between-individuals factors standardized loadings and correlations for the Evening PD-AID.

Global model fit and comparisons for the MFA models for the Morning and Evening PD-AID are presented in Tables 2, 3. Based on a scaled Satorra-Bentler χ^2^ test, the four within-individual factor solution was selected for the Morning PD-AID. Next, the four within-individual factor solution was carried into the between-individual factor models. The final solution for the Morning PD-AID was four within-individual factors and one between-individual factor (4W 1B). The between-individual factor included all 10 Morning PD-AID items (except the dichotomous item 9-Delay). The four within-individual factors provided separation by core physical actions (factor 1), basic self-care activities (factor 2), feeding (factor 3), and interference & dependence (factor 4). Using a similar procedure, for the Evening PD-AID, the final selected solution was five within-individual and four between-individual factors (5W 4B). The four between-individual factors identified basic ADLs (factor 1), life interference (factor 2), impact on planning (factor 3) and emotional consequences (factor 4). The five Evening PD-AID within-individual factors were basic ADLs (factor 1), toileting (factor 2), life interference (factor 3), impact on medication planning (factor 4), and emotional consequences (factor 5). Figures 1, 2 illustrate the factorial structure of the Morning and Evening PD-AID at the within- and between-individual levels. All factors had good to excellent reliability except for Evening PD-AID between factor 4 (Supplementary Table 4). The same factorial structure was identified using the initial dataset (with missing data) and the five datasets generated after multiple imputation.

**Table 2:** Models for the Morning PD-AID.

**Model**	**S-B** ** χ^2^**	**S-B** ** df**	**Scaled** ** Δ S-B** ** χ^2^**	**Scaled** ** Δ S-B** ** df**	**RMSEA [90% CI]**	**CFI**	**TLI**	**SRMRW**	**SRMRB**
1W Unrestricted B	100.66	26.71			0.036 [0.029, 0.042]	0.987	0.965	0.057	
2W Unrestricted B	49.59	19.75	66.07^**^	9	0.026 [0.018, 0.034]	0.995	9.981	0.046	
3W Unrestricted B	17.26	13.57	41.83^**^	8	0.011 [0.000, 0.023]	0.999	0.997	0.038	
4W Unrestricted B	4.67	9.60	22.21^*^	7	0.00 [0.000, 0.009]	1.000	1.000	0.024	
5W Unrestricted B	0.66	5.49	5.85	6	0.000	1.000	1.000	0.016	
4W 1B	5.16	10.63			0.000	1.000	1.000	0.024	0.026

** p < 0.001,

* p < 0.05 (W, Within-Individual; B, Between-Individual; S-B, Satorra-Bentler; RMSEA, Root Mean Square Error of Approximation; SRMR, Standardized Root Mean Squared Residual, CFI, Comparative Fit Index; TLI, Tucker-Lewis Index).

**Table 3:** Models for the Evening PD-AID.

**Model**	**S-B** ** χ^2^**	**S-B ** ** df**	**Scaled** ** ΔS-B** ** χ^2^**	**Scaled** ** ΔS-B** ** df**	**RMSEA [90% CI]**	**CFI**	**TLI**	**SRMRW**	**SRMRB**
1W Unrestricted B	509.47	97.63			0.046 [0.042, 0.050]	0.944	0.872	0.091	
2W Unrestricted B	215.83	79.16	238.50^**^	15	0.029 [0.025, 0.034]	0.981	0.948	0.073	
3W Unrestricted B	58.84	64.15	146.40^**^	14	0.000 [0.000, 0.011]	1.000	1.000	0.041	
4W Unrestricted B	31.20	52.13	29.16^*^	13	0.000	1.000	1.000	0.041	
5W Unrestricted B	14.47	43.95	24.52^*^	12	0.000	1.000	1.000	0.035	
5W 4B	14.52	50.53			0.000	1.000	1.000	0.035	0.012

** p < 0.001,

* p < 0.05 (W, Within-Individual; B, Between-Individual; S-B, Satorra-Bentler; RMSEA, Root Mean Square Error of Approximation; SRMR, Standardized Root Mean Squared Residual, CFI, Comparative Fit Index; TLI, Tucker-Lewis Index).

## Discussion

The aim of the current study was to identify the factor structure at within- and between-individual levels for the Morning and Evening PD-AID using data from 93 patients with a diagnosis of PD who completed at least one administration of either scale. Compliance with completion was high for both scales with an average of 78% of participants completing the Morning PD-AID and 74% completing the Evening PD-AID. An important finding was a high ICC (0.81 for Morning PD-AID and 0.83 for Evening PD-AID), demonstrating very good reliability of measurement when the Morning and Evening PD-AID were used for ~28 days. Further, ICCs were 0.75 for the Morning PD-AID and 0.78 for the Evening PD-AID when completed for one week (first week of the study). These results indicate that the Morning and Evening PD-AID can be used for pre-defined target assessment weeks spaced across several months during a clinical trial.

The MFA identified different factor structures for the within- and between-individual covariance matrices across the Morning and Evening PD-AID groups of items. For the Morning PD-AID, four within-individual and one between-individual factors were identified. For the Evening PD-AID, a structure including five within-and four between-individual factors provided the best model for the data. Within-individual factor 2 for the Evening PD-AID (toileting) containing a single item is reported for completeness and its conceptual relevance; however, factor analysis guidelines recommend elimination of such factors due to lack of reliability. Whether this factor is retained in the final structure and scoring recommendations will depend upon results of advanced psychometrics methods based on Item Response Theory. The factorial structure for both the Morning and Evening PD-AID mirrored the five conceptual domains (core physical actions, basic self-care activities, other daily activities, social impacts, and emotional impact) identified during the concept exploration interviews and reported in the conceptual model of PD-AID (5). The differences in factor structures, as well as loadings (items' ability to discriminate at within-versus between-individual level of analysis) stress the importance of evaluating factor validity at multiple levels when using longitudinal data collection instruments such as the PD-AID. The results have implications for developing scoring algorithms tailored to different contexts (i.e., clinical trials versus individual patient monitoring). In the context of a clinical trial, the emphasis is on between-individual differences (i.e., placebo versus active groups comparisons) while in a clinical patient monitoring context (e.g., when a follow-up is done with a patient) the within-individual variation is critical.

In the context of establishing the factor structure for the PD-AID, we employed the use of MFA. To the best of our knowledge, the current study represents the first use of MFA for validation of a PD clinical outcome assessment instrument.

Multiple limitations regarding the sample from this study have been described in a prior publication (13) and are relevant to these analyses as well. Participants had less advanced PD than initially planned likely due to the fact that patients with severe PD symptoms were less willing to participate because of the study length and burden of answering questions electronically on a daily basis. Although the sample size was small (93 participants), the use of longitudinal data in this sample provided a considerable sample for the MFA (2,175 datapoints for Morning PD-AID and 1,196 datapoints for Evening PD-AID).

While important in establishing the factor structure of the PD-AID using longitudinal data, our analysis has the additional limitation that the factor structure was identified based on an exploratory MFA and not cross-validated using a confirmatory MFA in a different sample. Because the sample size of the original study was relatively small, we could not create two random sub-samples to conduct the exploratory analysis on one sample, followed by confirmatory analysis on the second one. Replication of the current factor structure on a different sample using a confirmatory MFA will be important to establish the dimensionality invariance of the PD-AID. Further, due to the exploratory nature of our analysis, no covariates were considered. The use of a confirmatory MFA in future studies will allow the inclusion of covariates (i.e., gender, age, PD severity) using Multiple Indicators Multiple Causes (MIMIC) approach (25). The recommended factor scores are based on classical test theory and an important next step is to use an Item Response Theory approach to model the relations between the items and the underlying latent variables. Our final PD-AID structure and scoring recommendations will be complete after employment of modern test theory methods and will be informed by the findings from the analyses presented here. Once complete, PD-AID subscales will be named to facilitate outcomes investigations at both the full scale and subscale level. Further, such an approach will increase the precision of measurement using the PD-AID.

## Conclusions

The methods and results presented are an important component of the evaluation of fit for purpose (FFP) evidence exploring the structure of the PD-AID. While other patient-reported measures for PD are available, the PD-AID is the first to fully employ and document the methodologies laid out in the FDA Patient-Focused Drug Development (PFDD) Guidance (26–28) for a well-developed and fit-for-purpose clinical outcomes assessment. Prior publications have presented the PD-AID's qualitative content validity evidence and the initial classical test theory validation results (5, 13). The remaining effort to finalize the PD-AID structure and scoring recommendations utilizing modern test theory methods is forthcoming. The current study utilizing the full potential of the longitudinal data demonstrates the multifactorial structure of the PD-AID at both within- and between-individual levels and suggests the latent variables that will be used in the forthcoming Item Response Theory modeling. Documenting and publishing the full body of PFDD FFP evidence for the PD-AID will serve drug developers targeting treatments that represent what matters to individuals living with moderate to advanced PD who experience motor fluctuations.

## Data availability statement

The raw data supporting the conclusions of this article will be made available by the authors, without undue reservation.

## Ethics statement

The studies involving human participants were reviewed and approved by Copernicus Group Independent Review Board (IRB) who provided ethics review for all but one recruitment site. For the remaining site, the University of Florida Institutional Review Board (IRB), facilitated by the National Parkinson Foundation (NPF) Centers of Excellence, was utilized. The patients/participants provided their written informed consent to participate in this study.

## Author contributions

LD: study conduct and data collection—conception, organization, and execution, statistical analysis—design, review and critique and manuscript preparation—review and critique. CS: statistical analysis—design, execution, review, and critique, writing of the first draft, and manuscript preparation—review and critique. BS: statistical analysis—design, review, and critique, and manuscript preparation—review and critique. CM: statistical analysis—design, review, and critique, and manuscript preparation—review and critique. All authors contributed to the article and approved the submitted version.

## Funding

The authors declare that this study received funding from Pfizer Inc. The funder had the following involvement with the study: Pfizer had no involvement in the study beyond the contributions of LSD, an employee of Pfizer at the time this research was conducted.

## Conflict of interest

CS, BS, and CM are employees of Cronos Clinical Consulting Services, Inc., the entity responsible for licensing of the PD-AID, including commercial license fees for use in industry-sponsored clinical trials. LD is the lead developer of the PD-AID and an employee of Pfizer, Inc., the funding source for the research. No authors receive any licensing fees from the PD-AID.

## Publisher's note

All claims expressed in this article are solely those of the authors and do not necessarily represent those of their affiliated organizations, or those of the publisher, the editors and the reviewers. Any product that may be evaluated in this article, or claim that may be made by its manufacturer, is not guaranteed or endorsed by the publisher.

## References

[1] Williams DR Litvan I. Parkinsonian syndromes. Continuum. (2013) 19:1189–212. 10.1212/01.CON.0000436152.24038.e0 24092286 PMC4234134

[2] Dorsey ER Constantinescu JP Thompson KM Biglan RG Holloway K Kieburtz FJ. Projected number of people with Parkinson disease in the most populous nations, 2005 through 2030. Neurology. (2007) 68:384–6. 10.1212/01.wnl.0000247740.47667.03 17082464

[3] Thanvi B Lo N Robinson T. Levodopa-induced dyskinesia in Parkinson's disease: clinical features, pathogenesis, prevention and treatment. Postgrad Med J. (2007) 83:384–8. 10.1136/pgmj.2006.054759 17551069 PMC2600052

[4] Rizos A Martinez-Martin P Odin P Antonini A Kessel B Klemencic Kozul T. Characterizing motor and non-motor aspects of early-morning off periods in Parkinson's disease: An international multicenter study. Parkinsonism Relat Disord. (2014) 20:1231–5. 10.1016/j.parkreldis.2014.09.013 25269446

[5] Deal LS Flood E Myers DE Devine J Gray DL. The Parkinson's disease activities of daily living, interference, and dependence instrument. Mov Disord Clin Pract. (2019) 6:678–86. 10.1002/mdc3.12833 31745478 PMC6856450

[6] Guidance for Industry Patient-Reported Outcome Measures: Use in Medical Product Development to Support Labeling Claims Washington DC2009. Available online at: https://www.fda.gov/downloads/drugs/guidances/ucm193282.pdf (accessed April 12, 2022).

[7] Vizcarra JA Sánchez-Ferro Á Maetzler W Marsili L Zavala L Lang AE. The Parkinson's disease e-diary: developing a clinical and research tool for the digital age. Movement Disord. (2019) 34:676–81. 10.1002/mds.27673 30901492 PMC8114172

[8] Rendas-Baum R Buck PO White MK Castelli-Haley J. Psychometric validation of the revised SCOPA-diary card: expanding the measurement of non-motor symptoms in parkinson's disease. Health Qual Life Outcomes. (2011) 9:69. 10.1186/1477-7525-9-69 21851616 PMC3173285

[9] Banerji D Kulich K Keininger DL Tiplady B. Symptoms and impact of COPD assessed by an electronic diary in patients with moderate-to-severe COPD: psychometric results from the SHINE study. IntJ Chronic Obst Pulmonary Dis. (2015) 10:79–94. 10.2147/COPD.S73092 25609942 PMC4293297

[10] Muthén BO. Multilevel covariance structure analysis. Sociol. Methods Res. (1994) 22: 376–98. 10.1177/0049124194022003006

[11] Reise SP Ventura J Nuechterlein KH Kim KH. An illustration of multilevel factor analysis. J Personal Assess. (2005) 84:126–36. 10.1207/s15327752jpa8402_02 15799887

[12] Molenaar PC. A manifesto on psychology as idiographic science: Bringing the person back into scientific psychology, this time forever. Measurement. (2004) 2:201–18. 10.1207/s15366359mea0204_1

[13] Deal L Andrae DE Myers DE Johnson N Foster B Evans CJ. The measurement performance of the Parkinson's disease activities of daily living, interference and dependence instrument. Front Neurol. (2022) 13:760174. 10.3389/fneur.2022.760174 35432147 PMC9009412

[14] Hoehn MM Yahr MD. Parkinsonism: onset, progression and mortality. Neurology. (1967) 17:427–42. 10.1212/wnl.17.5.427 6067254

[15] SAS Institute Inc. SAS® 9.4 Statements: Reference. Cary, NC: SAS Institute Inc (2013).

[16] Muthén LK Muthén BO. Mplus: Statistical Analysis with Latent Variables: User's Guide (Version 8.5). Los Angeles, CA: Authors (2017).

[17] Terwee CB Bot SD de Boer MR van der Windt DA Knol DL Dekker J. Quality criteria were proposed for measurement properties of health status questionnaires. J Clin Epidemiol. (2007) 60:34–42. 10.1016/j.jclinepi.2006.03.012 17161752

[18] Black AC Harel O Matthews G. Techniques for modeling intensive longitudinal data with missing values. In: Conner TS, Mehl M, editors. Handbook of Research Methods for Modeling Daily Life. New York, NY: Guilford Press (2012). p. 339–56.

[19] Muthén BO. Multilevel factor analysis of class and student achievement components. J Educ Measur. (1991) 28:338–54. 10.1111/j.1745-3984.1991.tb00363.x

[20] Grilli L Rampichini C. Multilevel factor models for ordinal variables. Struct Equ Model Multidiscip J. (2007) 14:1–25. 10.1080/10705510709336734

[21] Satorra A Bentler PM. Ensuring positiveness of the scaled difference chi-square test statistic. Psychometrika. (2010) 75:243–8. 10.1007/s11336-009-9135-y 20640194 PMC2905175

[22] Hu LT Bentler PM. Cutoff criteria for fit indexes in covariance structure analysis: conventional criteria versus new alternatives. Struct Equ Model Multidiscip J. (1999) 6:1–55. 10.1080/10705519909540118

[23] McDonald RP. Test Theory: A Unified Treatment. Mahwah, NJ: Lawrence Erlbaum (1999).

[24] Streiner DL Norman GR. Health Measurement Scales: A Practical Guide to their Development and Use. 5th ed. Oxford: Oxford Univ Press (2008).

[25] Tsaousis I Sideridis GD Al-Harbi K. Examining differences in within-and between-person simple structures of an engineering qualification test using multilevel MIMIC structural equation modeling. Front Appl Math Stat. (2018) 4:3. 10.3389/fams.2018.00003

[26] United States Department of Health Human Services Food Drug Administration. Guidance for Industry Patient-Reported Outcome Measures: Use in Medical Product Development to Support Labeling Claims 2009 [Updated 2009; Cited 14th July 2021]. Available online at: https://www.fda.gov/media/77832/download (accessed June 14, 2022).

[27] United States Department of Health Human Services Food Drug Administration. Patient-Focused Drug Development: Collecting Comprehensive and Representative Input. (2020) [Updated 2020; Cited]. Available online at: https://www.fda.gov/media/139088/download (accessed June 14, 2022).

[28] Patient-Focused Drug Development Guidance Public Workshop. Methods to Identify What is Important to Patients and Select, Develop or Modify Fit-for-Purpose Clinical Outcomes Assessments. Workshop Date: October 15–16, 2018. Available online at: https://www.fda.gov/media/116277/download (accessed June 14, 2022).

