# Supplementary material for: Longitudinal investigation of the factor structure of the Parkinson's disease activities of daily living, interference and dependence instrument
Source: Front Neurol. 2022 Sep 23;13:941788. doi: 10.3389/fneur.2022.941788 (PMC9541427; doi:10.3389/fneur.2022.941788)
Supplement: Supplementary file 1 [file Data_Sheet_1.docx]

Supplementary Material

# Supplementary Figures and Tables

## Supplementary Tables

**Supplementary Table 1**. Average Percentage of Responses for the Morning PD-AID Across the Entire Study Duration (N=2175)

| **ITEMS** | **0**  **Not at all Difficult** | **1** | **2** | **3** | **4**  **Extremely Difficult** | **5**  **DONE with HELP** | **6**  **NOT DONE (Due to Parkinson’s)** | **NOT DONE**  **(Reasons Other than Parkinson’s)** |
| --- | --- | --- | --- | --- | --- | --- | --- | --- |
| **I1-Getting Out of Bed** | 55.3%  (1202) | 28.1%  (611) | 9.9%  (215) | 3.7%  (81) | 1%  (21) | 1.7%  (38) | 0.1%  (2) | 0.2%  (5) |
| **I2-Walking Around the House** | 56.6%  (1232) | 26.7%  (580) | 10.1%  (219) | 4%  (88) | 0.4%  (9) | 1.7%  (36) | 0.04%  (1) | 0.5%  (10) |
| **I3-On/Off Toilet** | 63.9%  (1390) | 21.1%  (458) | 8.3%  (181) | 2.9%  (63) | 0.04%  (1) | 1.4%  (31) | 0.1%  (2) | 2.3%  (49) |
| **I4-Taking a Shower** | 44.8%  (975) | 19.9%  (432) | 8.9%  (194) | 2.3%  (49) | 0.1%  (2) | 2.9%  (64) | 2.1%  (46) | 19%  (413) |
| **I5-Grooming Oneself** | 58.4%  (1274) | 26.9%  (587) | 8.3%  (181) | 2.6%  (57) | 0.04%  (1) | 1.1%  (25) | 0.5%  (11) | 2%  (44) |
| **I6-Dressing Oneself** | 53.1%  (1156) | 26.7%  (581) | 9.9%  (216) | 2.3%  (50) | 0.0%  (0) | 5.8%  (126) | 0.4%  (9) | 1.7%  (37) |
| **I7-Preparing Food/Drink** | 62.4%  (1357) | 22.1%  (480) | 5.4%  (117) | 2.3%  (50) | 0.3%  (7) | 3.7%  (80) | 0.6%  (14) | 3.2%  (70) |
| **I8-Feeding Oneself** | 67.5%  (1468) | 21.6%  (469) | 6.6%  (144) | 1.3%  (29) | 0.0%  (0) | 0.4%  (8) | 0.5%  (11) | 2.1%  (46) |
| **I9-Delay** | 82.8%  (1800)  **(NO)** | 17.2%  (375)  **(YES)** |  |  |  |  |  |  |
| **I10-Interference** | 39.7%  (864)  **(Not at all)** | 27.9%  (607) | 17.3%  (377) | 8.0%  (174) | 5.1%  (110) | 1.7%  (38) | 0.2%  (5)  **(Completely)** |  |
| **I11-Dependence** | 59.9%  (1302)  **(Not at all)** | 20.6%  (447) | 11.8%  (257) | 4.3%  (93) | 2.7%  (59) | 0.3%  (6) | 0.5%  (11)  **(Completely)** |  |

**Supplementary Table 2**. Average Percentage of Responses for the Evening PD-AID Across the Entire Study Duration (N=1996)

| **ITEMS** | **0**  **Not at all Difficult** | **1** | **2** | **3** | **4**  **Extremely Difficult** | **5**  **DONE with HELP** | **6**  **NOT DONE (Due to Parkinson’s)** | **NOT DONE**  **(Reasons Other than Parkinson’s)** |
| --- | --- | --- | --- | --- | --- | --- | --- | --- |
| **I1-Walking Around the House** | 53.5%  (1067) | 26.9%  (536) | 10.8%  (216) | 5.2%  (104) | 0.3%  (5) | 3.3%  (66) | 0.05%  (1) | 0.05%  (1) |
| **I1-On/Off Toilet** | 63.6%  (1270) | 23.1%  (461) | 8.1%  (161) | 3.2%  (64) | 0.3%  (5) | 1.2%  (24) | 0%  (0) | 0.6%  (11) |
| **I3-Prepare Food/Drink** | 56.1%  (1120) | 22.9%  (458) | 6.3%  (125) | 3.0%  (60) | 0.4%  (7) | 8.1%  (161) | 0.8%  (15) | 2.5%  (50) |
| **I4-Feeding Oneself** | 63.8%  (1274) | 24.5%  (491) | 7.8%  (155) | 2.4%  (48) | 0.05%  (1) | 0.9%  (18) | 0.2%  (3) | 0.3%  (6) |
| **I5-Getting In/Out Vehicle** | 46.3%  (922) | 22.2%  (442) | 10.4%  (208) | 2.7%  (53) | 0.05%  (1) | 7.3%  (146) | 1.5%  (30) | 9.5%  (190) |
| **I6-Using Computer** | 56.6%  (1130) | 23.9%  (478) | 7.0%  (140) | 2.4%  (48) | 0.1%  (2) | 7.3%  (145) | 0.1%  (2) | 2.6%  (51) |
| **I7-Working** | 3.5%  (70) | 4.8%  (95) | 0.8%  (16) | 0.3%  (6) | 0%  (0) | 0.1%  (2) | 25.7%  (513) | 64.8%  (1294) |
| **I8-Interference with Leisure** | 30.8%  (615)  **(Not at all)** | 25.9%  (517) | 18.5%  (369) | 11.4%  (227) | 7.6%  (151) | 5.1%  (101) | 0.8%  (16)  **(Completely)** |  |
| **I9-Dependence** | 47.7%  (952)  (**Not at all)** | 21.9%  (437) | 16%  (319) | 8.3%  (165) | 5.2%  (104) | 0.8%  (15) | 0.2%  (4)  **(Completely)** |  |
| **I10-Plan Around Medication Schedule** | 39.5%  (789)  **(Not at all)** | 27.7%  (552) | 14.7%  (293) | 7.7%  (154) | 5.9%  (117) | 3.5%  (70) | 1.1%  (21)  **(A lot)** |  |
| **I11-Plan Around Medication Wearing Off** | 39.7%  (793)  **(Not at all)** | 24.6%  (492) | 15.6%  (312) | 9.2%  (183) | 6.7%  (133) | 3.1%  (62) | 1.1%  (21)  **(A lot)** |  |
| **I12-Adjust Medication Schedule** | 49.5%  (989)  **(Not at all)** | 22.2%  (443) | 11.4%  (227) | 8.7%  (174) | 4.4%  (87) | 3.2%  (63) | 0.7%  (13)  **(A lot)** |  |
| **I13-Prevented from Activities** | 37.5%  (748)  **(Never)** | 23%  (459) | 15.4%  (307) | 9.6%  (191) | 8.0%  (160) | 5.9%  (117) | 0.7%  (14)  **(Always)** |  |
| **I14-Wait to Start Activities** | 45.4%  (907)  **(Never)** | 24%  (479) | 14.4%  (288) | 7.3%  (146) | 5.3%  (105) | 2.9%  (58) | 0.7%  (13)  **(Always)** |  |
| **I15-Stop or Take a Break** | 38.5%  (768)  **(Never)** | 24.7%  (493) | 16.4%  (327) | 10.9%  (217) | 6.5%  (130) | 2.6%  (52) | 0.5%  (9)  **(Always)** |  |
| **I16-Frustration** | 29.5%  (589)  **(Not at all)** | 24.1%  (482) | 16.2%  (323) | 10.2%  (204) | 10%  (199) | 6.7%  (133) | 3.3%  (66)  **(Extremely)** |  |
| **I17-Anxiety** | 36.9%  (736)  **(Not at all)** | 24%  (480) | 15.9%  (317) | 9.8%  (195) | 7.8%  (156) | 4.5%  (89) | 1.2%  (23)  **(Extremely)** |  |
| **I18-Confidence (Reversed)** | 16.2%  (323)  **(Not at all)** | 24.2%  (484) | 15.5%  (309) | 18.5%  (369) | 13.1%  (262) | 6.9%  (138) | 5.6%  (111)  **(Extremely)** |  |

| **ITEMS** | **Intraclass Correlation Coefficient (ICC)**  **(Entire Study Duration)** | **Intraclass Correlation Coefficient (ICC)**  **(Study Week 1)** |
| --- | --- | --- |
| **Morning**  PD-AID |  |  |
| **I1-Getting Out of Bed** | .79 | .74 |
| **I2-Walking Around the House** | .79 | .71 |
| **I3-On/Off Toilet** | .83 | .75 |
| **I4-Taking a Shower** | .84 | .80 |
| **I5-Grooming Oneself** | .78 | .73 |
| **I6-Dressing Oneself** | .83 | .80 |
| **I7-Preparing Food/Drink** | .80 | .75 |
| **I8-Feeding Oneself** | .82 | .77 |
| **I10-Interference** | .80 | .76 |
| **I11-Dependence** | .81 | .74 |
| **Average** | **.81** | **.75** |
| **Evening PD-AID** |  |  |
| **I1-Walking Around the House** | .85 | .77 |
| **I2-On/Off Toilet** | .87 | .86 |
| **I3-Prepare Food/Drink** | .83 | .76 |
| **I4-Feeding Oneself** | .85 | .71 |
| **I5-Getting In/Out Vehicle** | .87 | .79 |
| **I6-Using Computer** | .83 | .75 |
| **I8-Interference with Leisure** | .83 | .76 |
| **I9-Dependence** | .90 | .86 |
| **I10-Plan Around Medication Schedule** | .79 | .77 |
| **I11-Plan Around Medication Wearing Off** | .79 | .77 |
| **I12-Adjust Medication Schedule** | .71 | .63 |
| **I13-Prevented from Activities** | .84 | .82 |
| **I14-Wait to Start Activities** | .81 | .82 |
| **I15-Stop or Take a Break** | .80 | .76 |
| **I16-Frustration** | .84 | .84 |
| **I17-Anxiety** | .81 | .81 |
| **Average** | **.83** | **.78** |

**Supplementary Table 3**. Intraclass Correlation Coefficients for PD-AID Items for the Entire Study Duration and the First Study Week

**Supplementary Table 4**. Omega Reliability Coefficients for the Morning and Evening PD-AID Factors

| **Scale** | **Reliability Coefficient (**Ω**)** |
| --- | --- |
| **Morning** PD-AID |  |
| **Within Individual Factors** |  |
| - **Factor 1** | .92 |
| - **Factor 2** | .86 |
| - **Factor 3** | .70 |
| - **Factor 4** | .77 |
| **Between Individuals Factors** |  |
| - **Factor 1** | .99 |
| **Evening PD-AID** |  |
| **Within Individual Factors** |  |
| - **Factor 1** | .89 |
| - **Factor 2** | .76 |
| - **Factor 3** | .87 |
| - **Factor 4** | .98 |
| - **Factor 5** | .94 |
| **Between Individuals Factors** |  |
| - **Factor 1** | .93 |
| - **Factor 2** | .77 |
| - **Factor 3** | .89 |
| - **Factor 4** | .61 |

## 1.2 Supplementary Figures


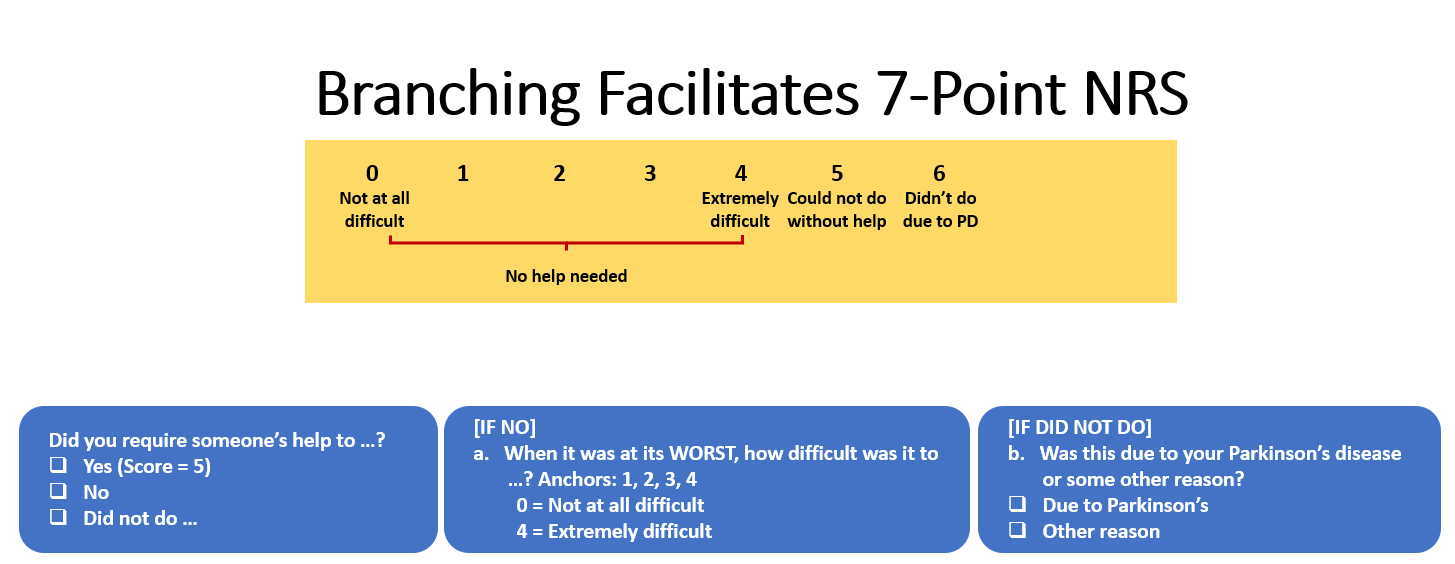


**Supplementary Figure 1.** Mapping of PD-AID items to a 7-point Numerical Rating Scale


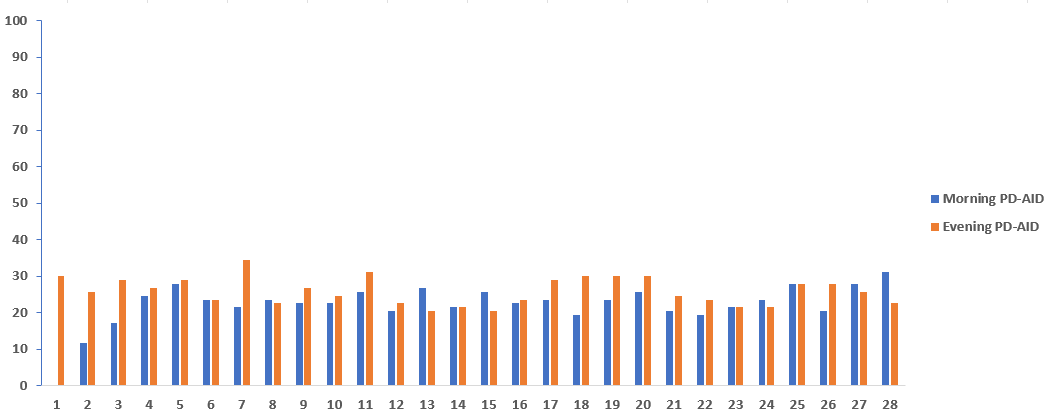


**Supplementary Figure 2.** Percentage of Missing Data Across 28 Study Days
